# Identification of a Novel *Acinetobacter baumannii* Phage-Derived Depolymerase and Its Therapeutic Application in Mice

**DOI:** 10.3389/fmicb.2020.01407

**Published:** 2020-07-21

**Authors:** Can Wang, Puyuan Li, Yang Zhu, Yong Huang, Mingming Gao, Xin Yuan, Wenkai Niu, Huiying Liu, Hang Fan, Yanhong Qin, Yigang Tong, Zhiqiang Mi, Changqing Bai

**Affiliations:** ^1^Department of Respiratory Medicine, Fuyang Hospital of Anhui Medical University, Anhui, China; ^2^Department of Respiratory and Critical Care Diseases, The Fifth Medical Center of Chinese PLA General Hospital, Beijing, China; ^3^State Key Laboratory of Pathogen and Biosecurity, Beijing Institute of Microbiology and Epidemiology, Beijing, China; ^4^Department of Respiratory and Critical Care Diseases, General Hospital of Shenzhen University, Shenzhen, China

**Keywords:** depolymerase, phage-derived, *Acinetobacter baumannii*, identification, therapeutic application

## Abstract

The rapid expansion of *Acinetobacter baumannii* clinical isolates exhibiting resistance to most or all available antibiotics is a global concern. Current treatments for infections caused by this bacterium have become less effective, and the need to explore new alternative therapies is urgent. Depolymerases derived from phages are emerging as attractive anti-virulence agents. In this study, a previously isolated *A. baumannii* phage (designated as vB_AbaM_IME285) was characterized, and genomic study was carried out using various bioinformatics tools. A gene predicted as encoding for the depolymerase was cloned and expressed, and the depolymerase activity of the recombinant enzyme (Dp49) was identified both *in vitro* and in experimental mice. The results showed that phage IME285 formed translucent halos around the plaques when inoculated onto a lawn of the host bacteria, exibiting depolymerase activity against this strain. On the basis of complete genome sequencing and bioinformatics analysis, ORF49 was speculated to be a gene encoding for the putative capsule depolymerase. The expressed recombinant Dp49 displayed an effective depolymerase activity and had a spectrum of activity similar to its parental phage IME285, which was active against 25 out of 49 *A. baumannii* strains. It was found that Dp49 greatly improved the inhibitory effect of serum on bacterial growth *in vitro*, and the administration of this enzyme significantly increased the survival rates of *A. baumannii*-infected mice in the animal experiment. In conclusion, the phage-encoded depolymerase Dp49 might be a promising alternative means of controlling infections mediated by multidrug-resistant *A. baumannii*.

## Introduction

*Acinetobacter baumannii (A. baumannii)* is a Gram-negative bacillus and is one of the main opportunistic pathogens of hospital-acquired infections. It often causes infections such as those of the respiratory tract, blood, urinary tract, wounds, and even the central nervous system (Perez et al., 2007). With the use of broad-spectrum antibiotics, immunosuppressants, and glucocorticoids, the multidrug-resistant *A. baumannii* (MDR-AB), pan-resistant *A. baumannii* (PDR-AB), and even carbapenem-resistant *A. baumannii* (CRAB) have emerged globally, and the treatment of infections caused by *A. baumannii* has become more difficult (Wong et al., 2017). Meanwhile, this organism is capable of continually spreading to new patients, making itself a nosocomial pathogen of particular clinical concern and a public health threat (Dijkshoorn et al., 2007). There is therefore an urgent need to develop new antimicrobial agents.

Bacteriophages, also known as phages, are viruses that could infect bacteria, and they are effective means for the treatment of drug-resistant infections (Chang et al., 2018). However, bacteria frequently live in biofilm communities surrounded by extracellular polymeric substances (EPS) that can act as a barrier to phage penetration (Azeredo and Sutherland, 2008; Pires et al., 2016a). It has been reported that EPS could protect bacteria against harsh external conditions and that they constitute an important survival strategy for bacteria (Pires et al., 2016b). For *A. baumannii*, EPS has increased the tolerance of the bacteria to antimicrobial agents due to diffusion limitations, potentially leading to severe persistent infections that are particularly difficult to treat, and this is also an important factor in resistance against phages (Hall-Stoodley et al., 2004). The thick capsular polysaccharide (CPS) surrounding Gram-negative bacteria is a crucial virulence factor in processes of infection and plays an important role in the phage-bacterium host interplay. By masking the receptors on the bacterial membrane, the potential for a phage to bind to the host cell is limited, so the infection ability of the bacteria is reduced (Samson et al., 2013). The unique ability of phage-derived depolymerases to specifically recognize and degrade CPS, EPS, and the O-antigen offers an attractive and promising tool for controlling pathogenic bacteria (Latka et al., 2017).

Our team has been working on the identification and application of phages and phage-derived proteins of Gram-negative bacteria for several years (Peng et al., 2014; Liu et al., 2016, 2019; Wang et al., 2016, 2019). In this research, a previously isolated phage was characterized, and its genome sequences were analyzed subsequently. ORF49, which was speculated as a gene encoding for the putative capsule depolymerase, was cloned and expressed *in vitro*, and its depolymerase activity against MDR-AB was testified. The enzyme greatly improved the ability of serum-mediated killing to counter bacterial growth and had promising therapeutic effects on *A. baumannii*-infected experimental mice.

## Materials and Methods

### *A. baumannii* Strain and the Isolation of Bacteriophage

Phage vB_AbaM_IME285, shortened as phage IME285, was isolated from untreated sewage in the fifth Medical Center of Chinese PLA General Hospital (Former 307th Hospital of PLA), and its host strain was *A. baumannii* Ab387 (stored in our lab). Firstly, the sewage from this hospital was centrifuged at 12,000 g for 10 min, and the supernatant was filtered through a 0.22-μm filter. By mixing 100 μL of the mid-exponential phase strain Ab387 (OD600 = 0.8) with 2 mL of 3× Luria-Bertani (LB) medium, the mixture was then added to 4 mL of filtered sewage. After a 2-h incubation at 37°C, the mixture was centrifuged and filtered again and was mixed with logarithmic phase strain Ab387. The mixture was poured onto double-layer agar LB plates, and clear phage plaques were picked after incubation for 6 h at 37°C. This procedure was repeated three times until a single clear plaque was selected. The phage was propagated by successive extended cultivation and purified using the cesium chloride gradient centrifugation method (Bachrach and Friedmann, 1971; Peng et al., 2014). Its titer was assessed by the double-layer agar method as described (Kropinski et al., 2009). Finally, this phage was titrated and designated as vB_AbaM_IME285 (short as IME285).

### Transmission Electron Microscopy and One-Step Growth Curve

A 25-μL purified phage IME285 suspension mixed with equal volumes of 4% paraformaldehyde and 0.5% glutaraldehyde was applied on carbon-coated copper grids and absorbed for 15 min. After removing the excess liquid, the phage mixture was stained with 2% phosphotungstic acid (PTA) for 30 min and subsequently dried, and then the phage particles were observed with a Philip TECNAI-10 transmission electron microscope (TEM) (Holland) (Wang et al., 2019).

Prior to one-step growth curve analysis, the multiplicity of infection (Samson et al., 2013) of phage IME285 was determined. An exponential growth phase culture of strain Ab387 was infected with 10-fold serial diluted solutions of phage IME285, and the mixtures were incubated on various LB plates for 2 hours at 37°C. The group with a rate of 10^–3^ resulted in the highest production of phage progeny (9.3 × 10^11^ pfu/mL), and this MOI was therefore chosen for subsequent experiments.

To assess the infection process of phage IME285, one-step growth curve experiments were performed as described previously (Wang et al., 2014). Briefly, phage IME285 was added to the strain Ab387 suspension, and then the mixture was spread on a double agar plate to determine the initial titer of the phage. Then, 100 μL phage solution (10^6^pfu/mL) was added to 1 mL (10^8^ cfu/mL) bacterial suspension (at an MOI of 0.001) and was incubated at 37°C for 5 min. After brief centrifugation (12,000 × *g*, 30 s), the pellets were washed twice and then resuspended in a final volume of 10 mL pre-heated LB broth before incubation. Samples collected at a 10-min interval for 150 min were diluted immediately, and the phage titration was performed on a double-layer agar plate against time to estimate the latent period and burst size (burst size = the total number of phages liberated at the end of one cycle of growth/the number of infected bacteria) (Gadagkar and Gopinathan, 1980). Tests were conducted three times.

### Whole-Genome Sequencing and Bioinformatics Analysis

The genomic DNA of phage IME285 was extracted using standard phenol/chloroform extraction protocols, as described previously, with minor modifications (Lu et al., 2013). The phage suspension was treated with 1 μg/mL of DNase I (Takara, Dalian, China) and RNase A (Takara, Dalian, China) at 37°C overnight to remove the bacteria nucleic acids and then inactivated at 80°C for 15 min. The sample mixed with a lysis buffer (final concentration 0.5% SDS, 20 mM EDTA, and 50 μg/mL proteinase K) was then incubated at 56°C for 1 h. Then, a standard phenol/chloroform extraction protocol was performed; the genomic DNA was mixed with an equal volume of isopropanol and was incubated for at least 2 h at −20°C. The mixture was centrifuged at 12000 × *g* for 10 min at 4°C. The pellets were washed twice with 75% ethanol and dissolved in nuclease-free water. Complete genome sequencing of phage IME285 was conducted using Illumina Miseq (San Diego, CA, United States), and all data were assembled using the Newbler v2.9 software, which was annotated online by RAST (Rapid Annotation using Subsystem Technology)^1^ (Margulies et al., 2005). The open reading frames (ORFs) were searched against the NCBI database^2^ using BLASTN^3^ and ORFfinder^4^. The sequence alignment of putative tail fiber proteins with a polysaccharide depolymerase domain, which is often located in the tail fiber or tail spike of a phage (Hernandez-Morales et al., 2018), was confirmed by using Easyfig_win_2.1 (Sullivan et al., 2011) with several related phages. The amino acid sequence alignment of the putative tail fiber proteins encoded by phage IME285 and three homologous phages was analyzed in CLC Genomics Workbench 8.0. A phylogenetic tree based on large terminal subunits with related phages from the NCBI database was generated using MEGA X software (Kumar et al., 2018).

### Protein Expression and Activity Verification

The target gene ORF49, which was predicted as encoding for the depolymerase of phage IME285, was amplified with the following primers: upstream primer ORF49-F: 5′-ATGACAAATCCAACACTTATTAC-3′ and downstream primer: ORF49-R: 5′-GGTTGGATATATTTGACCAGCTA-3′. The amplified fragment was cloned into the *pEASY^®^*-Blunt E1 expression vector with a N-terminal His × 6 tag (Beijing Trans Gen Biotech Co., Ltd., China). By verifying using the 3730*xl* DNA Analyzer (Thermo Fisher Scientific), the recombinant plasmid was then transfected into *E. coli* BL21 (DE3) (TransGen Biotech, Beijing, China). Cells carrying recombinant plasmids were selected on LB agar plates containing 1 mg/mL Ampicillin (Sigma–Aldrich). When the recombinant isolates in the incubation were in the exponential growth phase, they were induced with 1 mM isopropyl-D-1-thiogalactopyranoside (IPTG, Sigma–Aldrich). The overnight cultured cells were centrifuged at 13,000 × *g* for 10 min at 4°C, resuspended in lysis buffer (50 mM NaH_2_PO_4_, 300 mM NaCl, pH 8.0), and sonicated on ice (8–10 cycles with 30-s pulse and 30-s pause). Then, the bacterial lysate was again centrifuged at 13,000 × *g* for 10 min at 4°C, and the supernatant was filtered through a 0.22-μm filter. Finally, the filtrate was purified through a Ni-NTA column (Sangon Biotech, Shanghai, China) and was eluted with five volumes of imidazole-containing buffer (50 mM NaH_2_PO_4_, 300 mM NaCl, 250 mM imidazole, pH 8.0) via a step gradient to renature the purified production. By dialyzing with small molecular-mass-cut off membrane (Viskase, Willowbrook, IL, United States) in pre-cooled dialyzate overnight at 4°C, the molecular weight of the recombinant enzyme was measured by sodium dodecyl sulfate polyacrylamide gel electrophoresis (SDS-PAGE, Thermo Fisher Scientific). The protein bands were visualized by staining the gels with Coomassie brilliant blue (Sigma-Aldrich). The concentration of Dp49 was quantified by using the Bradford Protein Assay Kit (Thermo Fisher Scientific).

The activity of Dp49 was determined using spot assay. Briefly, 200 μL of overnight-cultured strain Ab387 suspension was added into 4 mL of molten soft agar and was incubated for 3 h for bacterial lawn formation. A series of 2-fold dilutions of purified enzyme Dp49 (with an initial concentrate of 0.25 mg/mL) was then dropped onto the plates with bacterial lawns, and these were inoculated overnight to monitor the formation of semi-clear spots as a measure of enzymatic activity. In addition, the sensitivity of other A. baumannii isolates to Dp49 (0.25 mg/mL) was determined in single-spot assays.

### Multilocus Sequence Typing of *A. baumannii* Strains and Determination of Activity Spectrum of Depolymerase and the Lytic Spectrum of Phage IME285

A total of 49 *A. baumannii* strains isolated from clinical sputum samples were used for multilocus sequence typing (MLST) typing according to the *A. baumannii* MLST database^5^ (Bartual et al., 2005). They were cultured in LB broth at 37°C for 10–12 h. The seven housekeeping genes (cpn60, fsuA, gltA, pyrG, recA, rplB, and rpoB) were amplified for all the *A. baumannii* strains, and the assembled sequences were aligned by using BLAST to assign the allelic numbers and sequence types (STs). The results were then compared with the available alleles in the *A. baumannii* MLST (Pasteur) database.

The activity spectrum of depolymerase and the host range of phage IME285 was determined by double-layer agar plate assay as described previously (Kropinski et al., 2009). Plates containing mixtures of the phage and distinct A. baumannii strains were incubated for 6 h at 37°C, and plaque-forming units (PFU) were counted for each combination. Relative efficiency of plating (EOP) was calculated as the average PFU number of the phage on target bacteria divided by the average PFU number on host bacteria (Khan Mirzaei and Nilsson, 2015).

### Assay of Serum Killing Contributed by the Depolymerase

Bactericidal contribution assay was performed as previously described, with some modifications (Pan et al., 2015). Briefly, 10 μL (about 10^8^ cfu/mL) of overnight-cultured strain Ab387 was incubated with 180 μL of serum (inactivated or activated) from some healthy volunteers, and 10 μL (0.25 mg/mL) of purified depolymerase Dp49 was incubated with an equal volume of phosphate-buffered saline (PBS) as a control, both at 37°C for 1 h. The mixture was diluted with PBS, and 100-μL volumes of dilutions were plated on solid LB agar plates and cultured overnight; the viable bacterial counts were subsequently counted.

### Depolymerase Treatment for Mouse Infections

Six groups of female BALB/c mice (12 per group, 18–20 g, specific-pathogen-free) were purchased from Beijing Vital River Laboratory Animal Technology Co., Ltd. (Beijing, China). All of the animal experiments were approved by the Institutional Animal Welfare Committee of the Beijing Institute of Microbiology and were performed in accordance with the guidelines of the Animal Welfare Agency. Mice were kept in individual cages with sufficient food and water and were euthanized by CO2 asphyxiation at the end of the experiment.

Three groups of mice were administered with 200 μL (6 × 10^7^ cfu) of *A. baumannii* strain Ab387 suspended in PBS via intraperitoneal injection, and the rest of the groups were infected with the same dose of strain Ab220. Half an hour later, a dose of 50 μg (200 μL) Dp49 (D group), 200 μL of phage IME285 with an MOI of 10 (P group) (Wang et al., 2016), or an equal volume of PBS (Control group, also represented as the C group) was given to mice in the different groups, respectively. Survival rates over a 96-h period were analyzed using the Kaplan–Meier analysis with the log-rank test. For the bacterial counting assay, four mice infected with strain Ab387 or strain Ab220 in the control group were euthanized, and their organs, including liver, spleen, and lungs, were resected within 24 h of being in a moribund state or dead. These organs were weighed, and their tissues were homogenized in PBS, respectively. Serially diluted homogenate was coated on LB agar plates and incubated at 37°C for 24 h to determine bacterial counts. The amounts of bacteria in organs were represented as CFU/mL and CFU/g of tissue, respectively. Four surviving mice from the depolymerase or phage groups were also euthanized, and their organ tissues were homogenized and coated on plates for bacterial colony counting, simultaneously.

### Statistical Analysis

All experimental data are represented as mean ± SD. Independent Student’s *t*-test was utilized to compare two groups, and two-way analysis of variance ANOVA) was used to compare multiple groups. All analyses were performed and plotted using Prism 6 software (GraphPad Software, CA, United States). A value of *P* < 0.05 was considered statistically significant.

## Results

### Morphology and One-Step Growth Curve of Phage IME285

**FIGURE 1:**
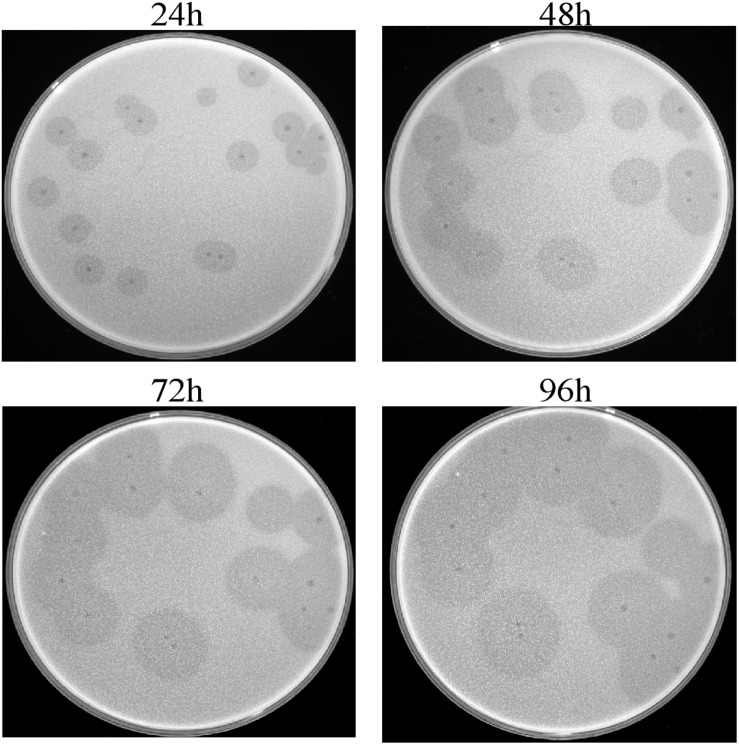
Plaques and halos produced by phage IME285. Clear plaques surrounded by translucent halos were observed on the lawns of host strain Ab387. The plaques and halos were photographed at 24, 48, 72, and 96 h, respectively. The translucent halos emerged at 24 h, and the area of the halos expanded with prolonged incubation.

**FIGURE 2:**
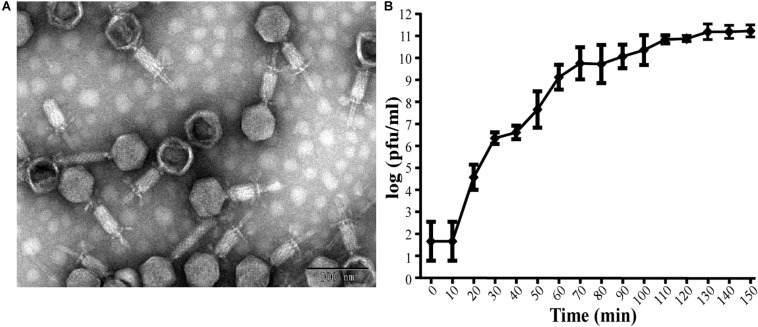
Transmission electron microscopy of phage IME285 and the one-step growth curve. **(A)** TEM of phage IME285. Purified phage particles of IME285 exhibited icosahedral heads and contractile tails. The bar represents a length of 100 nm. **(B)** One-step growth curve of phage IME285. The *x*-axis indicates the time post-infection, and the *y*-axis indicates phage titers. Each dot on the graph represents a mean titer. Phage IME285 had a latent period of approximately 10 min, an exponential growth period of 90 min, and a final plateau phase from 100 min onward. The estimated burst size of phage IME285 was 450 pfu/cell. The experiment was repeated three times, and results are expressed as mean ± SD.

This isolated phage could produce clear plaques surrounded by translucent halos (Figure 1) when inoculated onto a lawn of strain Ab387 bacteria (the antibiotic resistance profile is presented in Supplementary Tables S1, S2), and the area of the halos gradually increased as incubation progressed. Transmission electron micrographs showed that this phage has an icosahedral head (about 73 nm in diameter) and a long shrinkable tail (about 92 nm), which indicates that it belongs to the *Myoviridae* family (Figure 2A). It was therefore designated as vB_AbaM_IME285 according to the recommendations of the International Committee on Taxonomy of Viruses (ICTV) on phage nomenclature. Phage IME285 were propagated, concentrated, and purified to a final titer of 1 × 10^11^ pfu/mL. For one-step growth curve analysis, strain Ab387 was infected with phage IME285 at an MOI of 0.001. As shown in Figure 2B, the latency of phage IME285 was about 10 min, followed by a long exponential growth period of about 90 min, and it finally became stable, plateauing, after 100 min. The estimated burst size of phage IME285 was 450 pfu/cell.

### Whole-Genome Sequencing Analysis and Phylogenetic Tree

**FIGURE 3:**
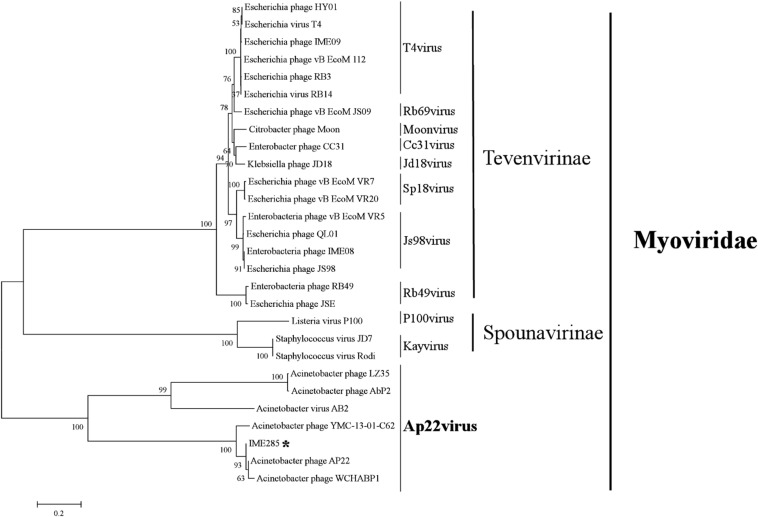
Phylogenetic tree of phage IME285. A phylogenetic tree based on the phage large terminal subunits from the reference strains was generated by the neighbor-joining method using the program MEGA X. The numbers next to the branches are bootstrap values and represent confidence (%). Phage IME285 belongs to the *Myoviridae* family, Obolenskvirus subclass.

**FIGURE 4:**
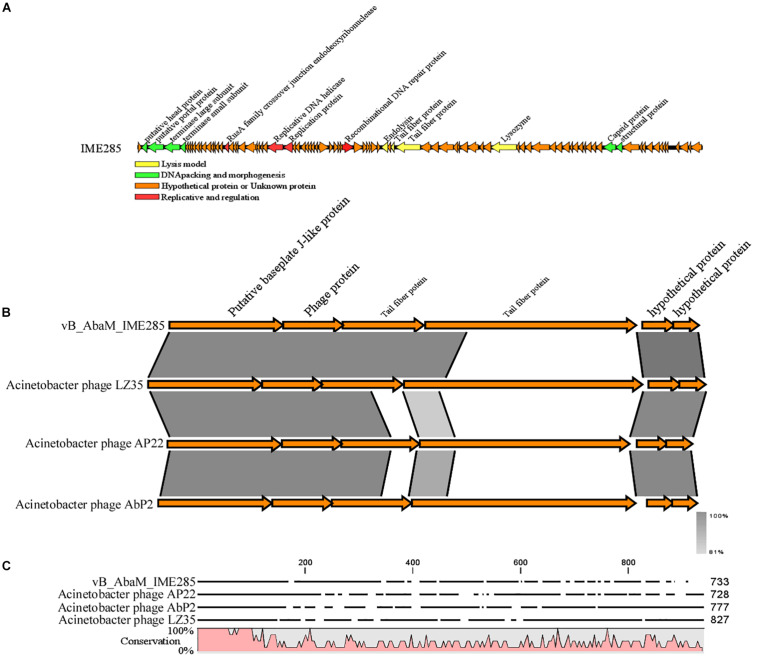
Annotation and features of predicted ORFs in phage IME285 genome, prior alignment of depolymerase sequences, and amino acid sequence alignment. **(A)** The annotation and features of predicted ORFs in phage IME285 genome. The genome of this phage encodes four kinds of functional proteins: lysis model, DNA packing and morphogenesis, hypothetical protein, and replicative model. **(B)** Linear alignment of the phage IME285 genomic sequence with Acinetobacter phage AbP2, AP22, and LZ35. Differences were observed in the tail fiber protein genes. **(C)** Amino acid sequence alignment of four putative tail fibers. The amino acid sequences in the C-terminal tail fiber regions of the four phages were significantly different, while the phage tail fiber proteins have a relatively short, highly conserved region (from 1 to 150 aa) at their N-terminal regions. High and low conservation of amino acid sequences is represented by 100 and 0%, respectively.

The complete genome of phage IME285 (GenBank Accession number: MH853786) consists of 45063 bp with double-stranded DNA, a low G + C content (37.9%), and a nucleotide content of 30.5% A, 31.6% T, 18.8% G, and 21% C. The annotation results of RAST indicated that the complete genome of phage IME285 contains putative 83 ORFs, predicted by NCBI BLASTP to encode four kinds of functional proteins, including the lysis model, DNA packing and morphogenesis, hypothetical protein, and replicative model (Figure 4A). A phylogenetic tree analysis based on the large terminal subunit revealed that this phage belongs to a subclass of the Obolenskvirus, which has demonstrated a very close relationship with *Acinetobacter* phage YMC-13-01-C62, *Acinetobacter* phage AP22, and *Acinetobacter* phage LZ35 from the *Myoviridae* family (Figure 3). BLASTp against the tail fiber amino acid sequence showed a high homology between phage IME285 and *Acinetobacter* phage AbP2 (accession number MF346584) (Query cover 16%, Ident 93.50%), *Acinetobacter* phage AP22 (accession number HE806280) (Query cover 14%, Ident 42.97%), and *Acinetobacter* phage LZ35 (accession number HE806280) (Query cover 16%, Ident 42.19%) (Figure 4B). Alignment based on amino acid sequence revealed that the tail fiber proteins of the four related phages shared a relatively short, highly conserved region (from 1 to 150 aa) at their N-terminal, while the C-terminal domains displayed obvious diversity, which might be a reason for the specificity of bacteriophages (Figure 4C).

### Expression, Purification, and Identification of the Recombinant Enzyme

**FIGURE 5:**
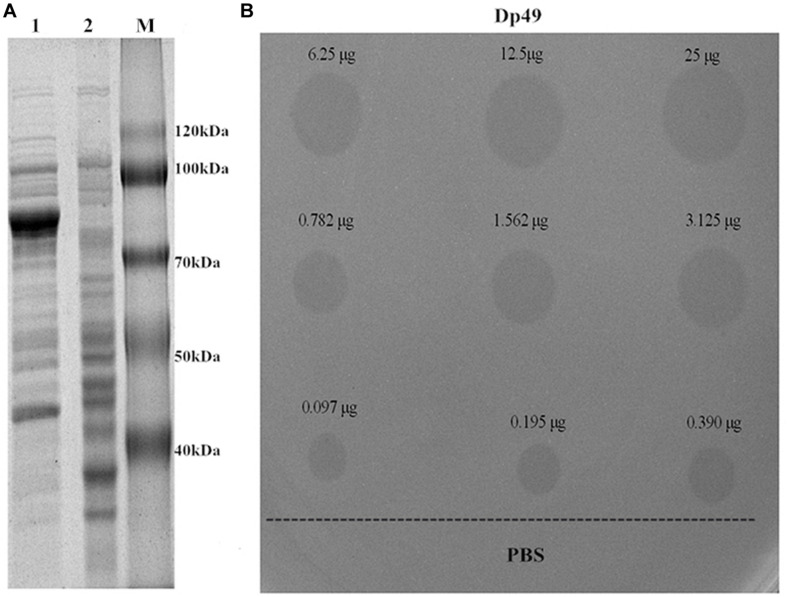
Depolymerase expression and activity. **(A)** SDS-PAGE analysis of expressed Dp49. Lanes: 1 is the total protein of the induced *E. coli* BL21 cells containing recombinant plasmid; 2 is the empty *E. coli* BL21 cells (control); M is the protein marker. The most abundant band is the target protein Dp49, with a predicted size of 78 kDa. **(B)** The determination of depolymerase activity. Serial dilutions (25–0.097 μg) of depolymerase Dp49 were dropped on the lawn of the host bacteria strain Ab387. The area of translucent halo reduced as the concentration decreased, and the halo disappeared when the enzyme was diluted to 0.097 μg. PBS was used as a negative control.

The target gene ORF49 encoding for the tail fiber protein was cloned into the expression vector, and the recombinant expression product was purified by the Ni-NTA column and dialysis. When analyzed with SDS-PAGE, a 78-kDa recombinant protein named Dp49 was obtained as predicted (Figure 5A). The activity of the purified enzyme was testified by spot assay. It showed that spotting with the purified recombinant protein could form a translucent halo on the lawn of the host bacteria strain Ab387 (Figure 5B). The area of the translucent halo reduced with decreasing Dp49 concentration until the enzyme was diluted to 0.097 μg, when the halo disappeared. The recombinant Dp49 exhibited good depolymerase activity.

### MLST of *A. baumannii* Strains, Activity Spectrum of Dp49, and Lytic Spectrum of Phage IME285

Results of the MLST analysis revealed that 42 *A. baumannii* strains were clustered into four undivided types (ST2, ST36, ST768, and ST268), whereas seven other strains were untypeable (Table 1). These bacterial strains were used to determine the depolymerase ability of the expressed depolymerase Dp49, and the lytic spectrum of phage IME285 was testified simultaneously. Thirty-nine strains, including strain Ab387 infected by phage IME285, all belonged to the ST2 type, and this was the dominant *A. baumannii* bacterial population. Moreover, 23 strains of this type were also sensitive to Dp49, which had the same spectrum of activity as did phage IME285 but seemed with a high sensitivity to depolymerase than to the phage. In total, more than half of these *A. baumannii* strains (26/49, 53.1%) could be depolymerized by Dp49, while less than a quarter (9/49, 18.4%) of them could be lysed by the phage IME285.

**TABLE 1:** List of *A. baumannii* strains used in this research and their ST results, including sensitivity to phage IME285 and depolymerase Dp49.

MLST type	Bacterial isolates	Sensitivity to phage	Sensitivity to depolymerase	Number of isolates
ST2	Ab14, Ab178, Ab406, Ab387, Ab7	+	+	5
	Ab2, Ab4, Ab5, Ab41, Ab1610, Ab1611, Ab1613, Ab1614, Ab2401, Ab2035, Ab2036, Ab2037, Ab2039, Ab1702, Ab1033, Ab1041, Ab1685, Ab1688, Ab1695	−	+	19
	Ab358, Ab363, Ab2529, Ab2589	+	−	4
	Ab333, Ab2712, Ab2093, Ab2099, Ab1697, Ab1706, Ab1058, Ab1689, Ab1692, Ab1694, Ab1697	−	−	11
ST36	Ab220	−	+	1
ST768	Ab 295	−	−	1
ST248	Ab 1612	−	−	1
NT^a^	Ab 2038	−	+	1
NT	Ab1704, Ab2114, Ab2116, Ab2126, Ab2129, Ab1707	−	−	6

Total				49

*^*a*^NT, not typeable; −, non-sensitive; +, sensitive.*

### Depolymerase Enhanced the Bactericidal Action of Serum Killing

**FIGURE 6:**
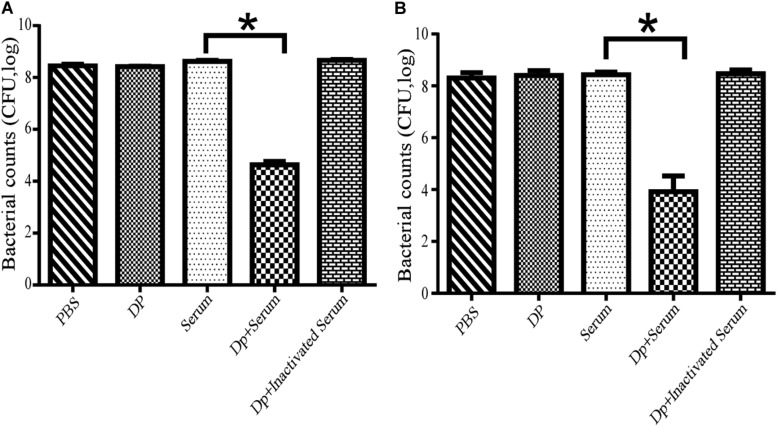
Serum killing assay mediated by Dp49. **(A)** Dp49 enhanced serum sensitivity of strain Ab387. **(B)** Dp49 enhanced serum sensitivity of strain Ab220. In the presence of active serum, Dp49 increased serum killing to both the bacteria Ab387 and Ab220, whereas in the presence of inactivated serum, Dp49, or PBS alone, the depolymerase showed no obvious effect on bacterial count. Serum alone showed a slight effect in killing. For Ab220 bacteria, Dp49 plus serum decreased bacterial loads by at least an order of 4 (Figure 6B). Three independent experiments were performed, and data are expressed as the counts of bacterial reduction (mean ± SD), and the bacterial counts of the two groups (serum and enzyme with serum) were compared by Student’s *t*-test (*P < 0.01).

The effects of serum killing after depolymerase treatment were evaluated *in vitro* against two *A. baumannii* strains, Ab387 and Ab220; of which the former was sensitive to both phage IME285 and the depolymerase, while the latter was only sensitive to the depolymerase. It showed that when the depolymerase or serum was applied separately, no bactericidal effect was observed in the bacterial counts. When combined with active serum, the bacterial counts of depolymerase-treated strain Ab387 and strain Ab220 all significantly decreased (*P* < 0.01) (Figure 6), and a maximum of approximately 10^4^ bacteria were killed (Figure 6B). However, when incubated with the inactivated serum, the bacterial counts scarcely reduced.

### Therapeutic Effect of Depolymerase in Mice

**FIGURE 7:**
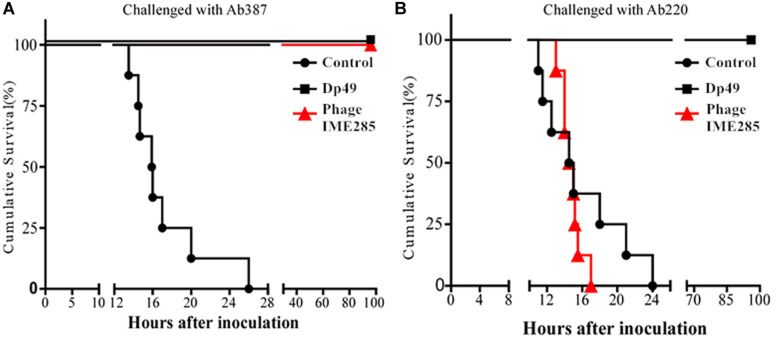
Therapeutic efficacy of depolymerase in mice. **(A)** Survival curves of mice infected with Ab387 after treatment. **(B)** Survival curves of mice infected with Ab220 after treatment. BALB/c mice were infected with 6 × 10^7^ cfu bacteria and treated with 50 μg (200 μL) depolymerase and an equal volume of phage IME285 or PBS half an hour later. The x-axis represents the time of infection in mice, and the y-axis represents the survival rate of mice. Statistical analysis was performed using the Kaplan-Meier method. Treatment with Dp49 (P < 0.0001; log–rank test) significantly increased the survival of mice infected with strain Ab387 or strain Ab220 over a 96-hour period.

To evaluate the therapeutic effect of depolymerase, mice were administered with 200 μL Dp49 half an hour post challenge with *A. baumannii* strain Ab387 or strain Ab220 in the experimental groups, while equal volumes of phage IME285 or PBS were administered in the rest of the groups. All mice in the control groups infected with lethal doses of bacteria died within 24–26 h, whereas mice in the experimental groups treated with Dp49 all survived. In the phage IME285-treated groups, mice infected with strain Ab387 were all rescued, but mice infected with strain Ab220 all died (Figure 7). The survival rate of the mice infected with strain Ab387 or strain Ab220 but treated with depolymerase was 100% within the monitored time period (0–96 h).

**FIGURE 8:**
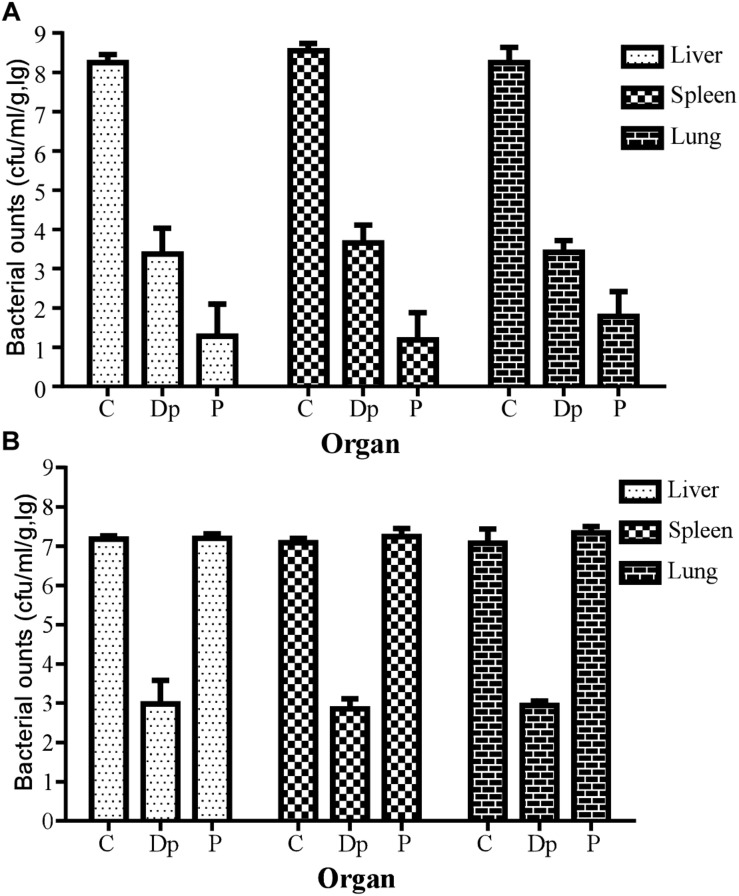
Measurement of tissue bacterial loads in multiple organs of infected mice. **(A)** Tissue bacterial loads of strain Ab387-infected mice after treatment. **(B)** Tissue bacterial loads of strain Ab220-infected mice after treatment. Bacterial loads in liver, spleen, and lungs of different treatment groups were measured. For bacteria Ab387 infection, bacterial loads were significantly reduced in the phage IME285-treated mice, followed by the Dp49-treated group. Meanwhile, tissue bacterial loads were decreased by more than 4 magnitudes approximately in Dp49-treated mice compared to others infected with strain Ab220. Data are expressed as mean ± SD. A regular two-way ANOVA test was conducted to analyze the differences in bacterial counts between the liver, spleen, and lungs (*P* < 0.05).

In a further study for bacterial enumeration, the organs of mice from the Dp49- or phage IME285-treated groups were collected and homogenized, and the organs of the control mice were also dissected in the near-death state. The results showed that the bacterial loads from organs such as liver, spleen, and lungs all significantly decreased in Dp49-treated groups (Figure 8). The degree of decrease in bacterial load of the different organs from mice treated with phage IME285 was more impressive compared to that of the Dp49-treated group when infected with strain Ab387 (Figure 8A). In contrast, phage IME285 showed little effect on bacterial elimination in mice infected with strain Ab220. Meanwhile, the bacterial count for Dp49-treated mice decreased by more than four orders of magnitude (Figure 8B).

## Discussion

Currently, antibiotic resistance in bacteria is a serious global problem, both medically and socially. Undoubtedly, antibiotics are still the most effective and reliable treatment option for bacterial infections, but the emergence of antibiotic-resistant bacteria such as MDR-AB and its rapid spread in hospitals and communities has become a serious threat to public health all over the world (Tomczyk et al., 2019). New drugs are not being developed fast enough to keep ahead of the natural ability of bacteria to evolve and defend themselves against antibiotics (Golkar et al., 2014). All of these problems indicate an urgent need to take further effective measures to control the abuse of antibiotics and to develop alternative antimicrobial agents (Brussow, 2017).

Bacteriophages (phages), which are natural enemies of bacteria, have the potential to be considered as an alternative strategy for treating infections mediated by multidrug-resistant pathogens (Kakasis and Panitsa, 2019). The main advantage of using bacteriophages as treatment options is their specificity, which can be designed to target pathogenic bacteria specifically without negatively affecting the normal microbiota and which do not cause secondary infections (Viertel et al., 2014). However, the high specificity of phages is a double-edged sword, as it means that the phage is also highly specific to its host bacteria, meaning that one single phage cannot cover the range of bacterial strains causing clinical infections. In our research, the lytic spectrum of phage IME285 was only 18.4% (9/49) against the 49 *A. baumannii* strains, which is not wide, after all.

A number of strategies have been designed to overcome this shortcoming, such as mixing several phages as a cocktail or in conjunction with antibiotics or developing a phage-derived enzyme-based complementary treatment. The fact that phage tail spikes or fibers usually exhibit depolymerase activity has been well illustrated (Pires et al., 2016b). Their great ability to degrade the thick capsule surrounding the cell wall of Gram-negative bacteria makes the depolymerases an attractive option for dealing with antibiotic-resistant bacterial infections. In this research, we identified a depolymerase, named Dp49, from a previously isolated phage, vB_AbaM_IME285. The bioinformatics analysis data indicated that gene ORF49 of the genome was predicted as the tail fiber protein. The expressed enzyme could form a translucent halo when inoculated onto a lawn of the host bacteria strain Ab387 *in vitro* and exhibited good polysaccharide depolymerase activity in spot assay. The results regarding the activity spectrum of this depolymerase showed that more bacterial strains exhibited sensitivity to Dp49 than to phage IME285. Interestingly, the majority of these Dp49-sensitive strains were clustered into ST2 according to Pasteur’s MLST. Capsular polysaccharide, also termed KL-type, is a major virulence factor for the *Acinetobacter* species. The depolymerases associated with capsular-targeting phage particles reduce the viscosity of CPS and strip it from the surface of encapsulated bacteria (Grażyna et al., 2016), and they have recently been considered as potential alternative anti-bacterial agents. Therefore, the identification of a certain KL-type-specific depolymerase makes sense. We compared the sequences of Dp49 with two KL-9-type depolymerases from phages AM24 (APD20249.1) and vB_AbaP_B5 (ASN73455.2) (Popova et al., 2019), and a high identity was recognized. The strains susceptible to Dp49 and IME285 exactly belong to the KL-9 capsular polysaccharide type. The KL type of the rest of the strains will be explored in subsequent research. Bactericidal contribution assay and a mouse therapy experiment mediated by Dp49 were performed to determine the capsule depolymerase activity of this phage-derived enzyme, and the safety of the phage-derived depolymerase was also evaluated. It was found that the phage-encoding enzyme Dp49 enhanced bacterial susceptibility to serum attack in the serum-killing experiments, leading to a 10^4^ reduction in the bacterial load. When treated with Dp49, it protected the mice from dying of lethal doses of bacterial infection. These findings agreed with some current research, and our results further confirmed that Dp49 showed a broader range and was more effective in the treatments compared with phage IME285. It is speculated that the removal or modification of bacterial surface structures, which are responsible for enhancing virulence, host recognition and colonization, and biofilm formation by pathogens, leads to a reduction in pathogenicity, bacteria sensitization against some antimicrobials, or host defenses such as phagocytosis by macrophages and the bactericidal action of serum (Bansal et al., 2014; Wang et al., 2016; Liu et al., 2019).

The unique ability of phage-derived depolymerases to specifically recognize and degrade CPS and EPS offers an attractive and promising tool for controlling pathogenic bacteria (Latka et al., 2017). Compared to phages, depolymerase is more convenient and flexible in application and is believed to have a better performance against bacterial biofilms. Furthermore, its use avoids some of the disadvantages of phages, such as purification and endotoxin removal. Depolymerases derived from phages can provide a new strategy for the treatment of multidrug-resistant bacteria and have attracted increasing interest as potential antimicrobial agents, particularly in light of emerging and spreading resistance of bacteria against classical antibiotics. However, the application of depolymerases against human infections still needs to be supported by further clinical trials.

## Conclusion

In this study, we identified a capsule depolymerase Dp49 derived from an *A. baumannii* phage. It greatly increased the inhibitory effect of serum on the growth of bacteria *in vitro* and could protect mice from dying of lethal doses of bacterial infection. This depolymerase might become a promising alternative strategy for controlling infections mediated by MDR-AB.

## Data Availability Statement

The datasets generated for this study can be found in the GenBank, Accession number: MH853786.

## Ethics Statement

The studies involving human participants were reviewed and approved by the Ethics Committee of the Fifth Medical Center of Chinese PLA General Hospital (Former 307th Hospital of PLA), and an exemption of informed consent was obtained (Ethics approval No. ky-2018-10-85).

## Author Contributions

CW and ZM isolated the phage and characterized its biological characteristics and also identified, expressed, and evaluated the depolymerase activity. YH, HF, HL, and MG performed the genome-wide sequencing and genomic analysis. CW, XY, WN, and YQ conducted the animal experiments. PL and CW drafted the manuscript. YT, ZM, and CB conceived and designed the experiments. All authors read and agreed to the publication of the manuscript.

## Conflict of Interest

The authors declare that the research was conducted in the absence of any commercial or financial relationships that could be construed as a potential conflict of interest.

## Footnotes

1 http://rast.nmpdr.org/

2 http://www.ncbi.nlm.nih.gov/

3 https://blast.ncbi.nlm.nih.gov/Blast.cgi

4 https://www.ncbi.nlm.nih.gov/orffinder/

5 http://pubmlst.org/abaumammii
