# Supplementary material for: Identification of a Novel Acinetobacter baumannii Phage-Derived Depolymerase and Its Therapeutic Application in Mice
Source: Front Microbiol. 2020 Jul 21;11:1407. doi: 10.3389/fmicb.2020.01407 (PMC7396526; doi:10.3389/fmicb.2020.01407)
Supplement: Supplementary file 1 [file Table_1.docx]

**Identification of a novel *Acinetobacter baumannii* phage-derived depolymerase and its therapeutic application in mice**

**Supplementary material**

Table S1. Drug sensitivity data of Ab220

| Drug | MIC(ug/ml) | sensitivity | Drug | MIC(ug/ml) | sensitivity |
| --- | --- | --- | --- | --- | --- |
| Amoxicillin and Clavulanate Potassium | ≤ 0.5 | S | Azithromycin |  | R |
| Gentamicin | ≤ 0.5 | S | Ampicillin sulbactam |  | S |
| imipenem | 0.25 | S | Ampicillin sulbactam |  | R |
| Oxacillin | 0.5 | S | Nitrofurantoin | 32 | S |
| rifampicin | ≤ 0.5 | S | Linezolid | 2 | S |
| tetracycline | ≤ 1 | S | Moxifloxacin | ≤ 0.25 | S |
| Cotrimoxazole | ≤ 10 | S | Temeticillin | ≤ 0.12 | S |
| vancomycin | ≤ 0.5 | S | Cefoxitin |  | I |
| Levofloxacin | ≤ 0.12 | S | Cefaclor |  | S |
| Cefotaxime |  | S | ceftriaxone |  | S |
| cefuroxime |  | S | Ciprofloxacin | ≤ 0.5 | S |
| Erythromycin | ≤ 0.25 | R | Clindamycin | ≥ 8 | R |

Table S2. Drug sensitivity data of Ab387

| Drug | MIC(ug/ml) | sensitivity | Drug | MIC(ug/ml) | sensitivity |
| --- | --- | --- | --- | --- | --- |
| Amikacin | ≤ 2 | S | Aztreonam | ≥ 64 | R |
| Gentamicin | ≥16 | R | Cefazolin | ≥ 64 | R |
| imipenem | ≤ 1 | S | Cefepime | 2 | S |
| meropenem | ≤ 0.25 | S | cefotetan | ≤ 4 | S |
| cefuroxime | ≥ 64 | R | Ceftazidime | 4 | S |
| Ciprofloxacin | ≥ 4 | R | ceftriaxone | ≥ 64 | R |
| Piperacillin Tazobactam | ≤ 4 | S | Tobramycin | ≥ 16 | R |
| Cotrimoxazole | ≤ 20 | S | Ampicillin sulbactam | 16 | I |
| Levofloxacin | ≥ 8 | R | Nitrofurantoi | ≤ 16 | S |
